# An integrative review to identify how nurses practicing in inpatient specialist palliative care units uphold the values of nursing

**DOI:** 10.1186/s12904-021-00810-6

**Published:** 2021-07-16

**Authors:** Sue Moran, Maria Bailey, Owen Doody

**Affiliations:** 1 Milford Care Centre, Castletroy, Limerick, V94 H795 Ireland; 2 Department of Nursing and Midwifery, Faculty of Education and Health Sciences, Health Research Institute, University of Limerick, Limerick, V94 T9PX Ireland

**Keywords:** Nursing, Palliative care, Integrative review, Values

## Abstract

**Background:**

Caring for individuals and their families with a life-limiting, symptomatic illness and those who are dying has long been an integral role of palliative care nurses. Yet, over the last two decades, the specialty of palliative care has undergone significant changes in technology and medical treatments which have altered both the disease trajectory and the delivery of palliative care. To date, there is little evidence as to the impact of these medical and nursing advancements on the role of nurses working in palliative care and how in clinical practice these nurses continue to uphold their nursing values and the philosophy of palliative care.

**Methods:**

An integrative review was conducted searching seven academic databases from the time period of January 2010 – December 2019 for studies identifying research relating to the role of the palliative care nurse working in specialist palliative care units and hospices. Research articles identified were screened against the inclusion criteria. Data extraction was completed on all included studies and the Crowe Critical Appraisal Tool was utilized to appraise the methodological quality and thematic analysis was performed guided by Braun and Clarke’s framework. The review was conducted and reported in lines with PRISMA guidelines.

**Results:**

The search yielded 22,828 articles of which 7 were included for appraisal and review. Four themes were identified: (1) enhancing patient-centred care (2) being there (3) exposure to suffering and death (4) nursing values seen but not heard. The findings highlight that while palliative care nurses do not articulate their nurse values, their actions and behaviors evident within the literature demonstrate care, compassion, and commitment.

**Conclusion:**

These findings suggest that there is a need for nurses working in specialist palliative care units to articulate, document, and audit how they incorporate the values of nursing into their practice. This is pivotal not only for the future of palliative nursing within hospice and specialist palliative care units but also to the future of palliative care itself. To make visible the values of nursing further practice-based education and research is required.

**Supplementary Information:**

The online version contains supplementary material available at 10.1186/s12904-021-00810-6.

## Background

Palliative care (PC) was initiated by Cecily Saunders in the United Kingdom as an alternative to the predominating biomedical technological approach to death and dying in hospitals that prevailed at that time [1]. Through Saunders work in the development of the modern hospice movement, a philosophy of care for the dying that was distinct from the hospital setting was developed [1]. In the ensuing years, PC has grown and developed internationally. In response to this growth, the World Health Organisation (WHO) has defined and redefined their position paper on palliative care [2–4]. These changes in the definitions over the years 1990 to 2020 both alter the future path of PC and reflect care provision. Palliative care is an approach that improves the quality of life of patients (adults and children) and their families who are facing problems associated with life-threatening illness. It prevents and relieves suffering through the early identification, correct assessment and treatment of pain and other problems, whether physical, psychosocial or spiritual [4]. In line with these changes and the developments in palliative medicine, palliative nursing has been required to keep pace with such changes while still upholding the essence of nursing care.

End of life care is a component embedded within PC [1] and it has been well documented that before the emergence of the modern hospice movement, end of life care was largely a nursing domain with scant recognition from the medical field [1]. Early modern hospices with a focus on comfort and care rather than cure meant that hospice nurses became central to the provision of such care. Such was the situation until the late twentieth century when in tandem with significant developments in diagnosis and treatments, PC became a medical specialty and a consultant-led multidisciplinary team providing patients with increased opportunities for improved symptom management [1]. Thus PC transitioned from being a nurse-led model to a bio-medical model and Saunders philosophy was recognized as applicable in all areas where there is a person with PC needs. In the ensuing years in line with continued scientific advancements a dichotomy developed between an increasing biomedical focus and that of the Askelpian tradition of healing and the early vision of PC [5]. This dichotomy between the peace and tranquility vision of PC and that of a busy specialist palliative care unit (SPCU), where the biomedical focus is in danger of overshadowing and undervaluing nursing care which values and focuses on the person who is confronting death and supporting them and those close to them.

Nursing places value in patient/family care as the core of nursing and the recurring values of comfort, kindness, dignity, commitment, and competency are seen as fundamental [6–8]. The unique role that nurses play in PC has evolved significantly as an “art” of nursing with nursing skills based on compassion, empathy, and genuine kindness which are given equal measure to that of the science of nursing [9]. Nursing values are part of the nursing profession and provide a framework to guide nurses’ goals, behaviors, and actions [8]. However, challenges in sustaining nursing values within the current dominating biomedical model of PC has the potential to create a tension between the art and science of nursing [10] and therefore compromising the values of nursing. This tension arises as while the activities that constitute the art of nursing are undertaken in all care actions/activities they are less visible and measurable than that of the science activities. This is important given that nursing codes of practice and models promote holistic care as integral to the nurses’ role and nursing values have always been considered the essence of nursing, driving patient care [11].

However, in recent years several reports [12–17] have identified deficiencies in nursing practice where patients have experienced suboptimal nursing. These findings and other similar reports have led to the reaffirming of nursing values resulting in these being revisited [18–20]. This focus on nursing values created an opportunity to refocus and address the imbalance that had occurred between physical disease and biomedicine and the emotional, psychological, spiritual wellbeing and healing relationship between patient and nurse [19, 21]. This imbalance needs to be considered in terms of the broader history of nursing and its perception. For example, historically it was considered that caring for the sick was a selfless unskilled vocation and the work of women. This led to a questioning of the worth of nurses and a desire to show accountability, competence, and articulation of levels of knowledge and specialized skills in the second half of the twentieth century. An influencing factor within this process was the sense of social worth and the holding of the scientific as a measure of worth. Thus, nursing strived to be more akin to other professions’ especially that of the medical profession. In tandem with this was professional advancement, where nurses became more focused on specialization and management roles, with less support for the affective role [22]. However, the issues of reported poor standards of care fuelled a focus on quality and excellence in professional standards, and achievement of such excellence demanded that quality be more firmly defined and more effectively measured, including the provision of compassionate care [22]. Considering this refocus on the values of nursing this paper presents a timely review of the PC literature of nursing values evidenced in the context of the changing face of PC.

## Methods

**Fig. 1:**
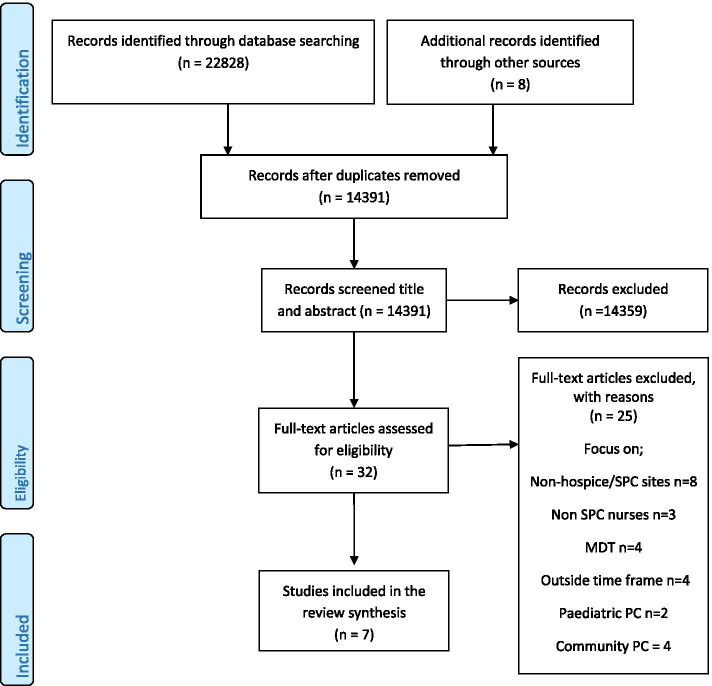
PRISMA 2009 Flow Diagram

This integrative review aimed to identify the values of nursing evidenced in palliative nursing. Kable et al.’s twelve step guide to searching and critiquing the research literature [23] was utilized to guide and support this review which is reported in line with the PRISMA checklist and flowchart [24] (Fig. 1 and Supplementary file 1).

### Search sources and strategies

Searches were performed in seven electronic databases: CINAHL, EMBASE, PsycINFO, AMED, Medline, Cochrane Library, and Lenus. The search strategy was based on the PEO (population, exposure, outcome) string framework. The following terms were included in the search string strategy; first, for population ‘palliative care nurs*’ ‘palliative care clinical nurs* specialist’ ‘palliative care nurs* specialist’ ‘hospice nurs*’ ‘specialist in palliative care’. Secondly, terms associated with exposure ‘end of life care facility’ ‘end of life care unit’ ‘hospice’ ‘specialist palliative care unit’ ‘specialist palliative care in-patient unit’. Thirdly, terms representing outcome ‘value’ ‘role’ ‘function’ ‘commitment’ ‘care’ ‘compassion’. All these terms were linked using the Boolean operator “OR” within each string and to combing search strings in each database “AND” were used.

### Criteria for considering studies in this review

#### Inclusion criteria

- The search was limited to peer-reviewed journals.
- Nurses working in inpatient hospice or specialist palliative care units.
- Articles are written in English or translation available.
- Articles referring to the adult population over 18.
- Articles published between 01 January 2010 and 01 December 2019.

#### Exclusion criteria

- Articles relating to persons under the age of 18 years.
- Nurse not working in inpatient hospice or specialist palliative care units.
- Studies with mixed samples where results related to PCNs cannot be extracted.
- Editorials, policies, conference proceedings*.*

### Study selection and data extraction

All retrieved results were exported to Endnote version X9 and duplicates were removed. Titles and abstracts of the remaining results were screened by the first author (SM) for eligibility against the inclusion criteria. Full text of all possible eligible papers were retrieved for further screening and the author team worked in pairs to make the final decision. Within this process reasons for excluding were documented and reported (Fig. 1). A total of 22,828 papers were retrieved and following the removal of duplicates 14,391 were screened. Papers were then removed based on title and abstract review from which 14,359 were removed leaving 32 papers for full-text screening. The 32 were then read in full-text and 25 were removed as they did not meet inclusion criteria leaving seven papers for inclusion in this review. Data were extracted from each of the seven papers by the lead author (SM) and reviewed by a second author (MB/OD). A data extraction table (Table 1) was developed and utilized to report; authors, year, title study aims; methods; sample size; nursing values evident; summary of findings; implications, and quality appraisal score: Crowe Critical Appraisal Tool (CCAT) [25].

**Table 1:** Data extraction table

**Author/s, Year, Title, Country**	**Study Aims**	**Methods**	**Sample Size**	**Nursing values evident**	**Summary of findings**	**Implications**	**CCAT Score**
Aeling and Chavez 2019 [34] Hospice care: Nurse’s experience and perception of older adult patients’ experience. USA	To understand the perspective of hospice nursing staff on the length of hospice stay as well as their perceptions of older adult experience.	Interpretative Phenomenological design. Semi structured interviews.	10 hospice nurses from 3 hospices.	Understanding what patients want when being cared for. Commitment to learning. Care/caring relevant to nursing practice. Compassion in nursing practice.	Patient related – Limited understanding of hospice and its role. Positive intact on longer length of stay. Enhances patient nurse relationship. Nurses related – Gets to know patient better /personalized care. Lack of knowledge of non-malignant. Late referrals. Less likely for crisis admission. Patient and staff related. More emotional support /more conservation round end of life.	Findings highlight - The importance of appreciated referrals. Education for patients on role of palliative care with non-malignant condition’s. Importance of patient -nurse relationship which facilitates personalized care. Developing practices that enhances nurse \patient relationship. Coaching /role modelling	90%
Balasubramanian and Read 2012 [28] Hospice nurses perceptions of caring for patients’ with a non-malignant diagnosis: a single site case study. UK	To explore nurses perceptions of caring for patients with non-malignant disease in a hospice setting	Qualitative research. Focus groups × 2	16 nurses.	Nurses wanting to care for non-malignant patients at end of life but not presently well prepared in some of knowledge/practice. Demonstrates compassion care commitment.	Patient relation – When to refer to hospice non cancer patients are experts in their care. Nurses related—Education re non- malignant patients some similar to cancer patients but some very different.	Education for patients and families re role of hospice. Education and skill preparation for nurses. Collaborative working practices. Hospice service development.	93%
Boa et al. 2018 [29] Patient centred goal setting in a hospice: a comparative case study of how health care practitioners understand and use goal setting in practice. Scotland	To investigate healthcare practitioners understanding and practice of patient centred goal setting in a hospice.	Mixed method comparative case study. Semi structured interview. Case note analysis. Observations	Doctors = 2. Nurses = 5. Physio = 1. OT = 1. SW = 1.	Developing models of practice to enhance patient care (care compassion commitment).	Goal setting is valued. Not consistent in practice. Missed opportunities for goal setting. More patient focused approach required. Challenges and other factors influence goal setting.	Goal setting in palliative care/which is part of ccc model.	100%
Ingebretsen et al. 2016 [32] Hospice nurses emotional challenges in their encounters with the dying. Denmark	To examine nurses emotional challenges when caring for the dying in a hospice setting.	A qualitative design using hermeneutic phenomenology. In depth interviews.	*N* = 10 nurses from 2 hospices using purposeful sampling.	Care Commitment and compassion in nursing practice (what it looks like in practice and nursing behaviours).	Nurses emotional touched by patients. Nurses identifying themselves with the patient and needing to distant themselves. Nurses balancing between their personal and professional being and caring for a dying patient. Reminds nurses of their own mortality, with leads to two both enriching.	Understanding the emotional element to nurses role. Development of education programmes for nurses to further the skills to manage both themselves and their patients in a compassionate and effective.	95%
Lavoie et al. 2013 [31] The integration of a person centred approach in palliative care. Canada	To document changes that occurred after the integration of a person centred approach focusing on human freedom, the human becoming school of thought.	Pre project –process and –post project descriptive qualitative design. Pre and post project phases consisted of collecting data from healthcare providers and relatives of patients through semi structured interviews.	51 health care provides 10 relatives Medical records of 10 patients.	Understanding the emotional work nurses adds to nursing knowledge/Carpers way of knowing, intuitive practice.	Following education sessions, the focus moved from tasks centred to person centre. Priority of respecting patients choices desires and needs. A presence shifting from being available to true listening. The affirmation of following the ever changing rhythm of the patient and a notion of respect.	Models for nursing practice /intuitive practice	93%
Powell et al. 2020 [30] Resilience in inpatient palliative care nursing: A qualitative systematic review. United Kingdom	To understand resilience from perspective of inpatient palliative care nurses.	Systematic Review—A thematic synthesis of qualitative studies. Spider Acronym informed the question. Data bases: Academic search ultimate, Cumulative index to nursing and allied health, Medline, Psycinfo, Scopus.	8 articles reviews.	Care and compassion in nursing practice.	Understanding stress in pc nursing/coping mechanisms. Strategies for emotional well being. Resilience occurs when nurses are able to include stressful aspects of their personal and professional lives into a coherent narratives.	Understanding the complexities of the nurses’ role. Development of effective coping mechanisms. Importance of debriefing, reflecting in and on practice. The role of clinical supervision/mentorship programmes.	100%
Vargas et al. 2013 [33] Redefining palliative care at a specialised care centre: A possible reality. Brazil	To describe how palliative care provided to patients in a SPC unit and how the nursing team works to preserve the unit as a whole.	Simple case study design. Semi structured interviews. Direct observations of nursing care.	5 nurses. 5 nursing technicians. Public teaching hospital.	Care and compassion in nursing practice.	A special place for people in a special situation. The goal of care is comfort and quality relationships between the patient/family and team. Environment issues – nice surroundings. Interventions that are not done. Patient centred care. Spiritual issues. Care modes. Palliative Care Unit. Importance of time. Pain control. Education with family/patient around needs. Touch, listening, being there, patient autonomy, communication.	Patient centred care.	75%

### Quality assessment of the included studies

The CCAT [25] in conjunction with its supporting user guide [26] was used to assess the quality of all included papers. CCAT enables the researcher to undertake a systematic and rigorous approach and is divided into eight categories consisting of preliminaries, introduction, design, sampling, data collection, ethical matters, results, and discussion. The methodological quality of the papers reviewed was good with CCAT scores varied amongst the papers ranging from 75 to 100% (30/40 to 40/40).

### Data analysis

Data analysis was guided by Braun and Clarke’s six-step thematic analysis inductive approach [27]. Initially, each study was carefully read and specific quotes and paragraphs relevant were highlighted. Open notes of early ideas and concepts were handwritten on all the papers. Following this an initial preliminary open coding of the entire data set was conducted to help streamline and converge the data. All concepts relevant to the research question were coded and a color-coding system highlighted related patterns across the research papers. This process was repeated for each paper and this enabled further familiarisation of the data and recurring patterns were also handwritten in a notebook for further reflection. Searching for themes, linking codes within and between papers facilitated the formation of broader, more conceptualized themes. The themes were then reviewed which involved combining and refining and rejecting some preliminary themes. Data difficult to categorize into distinct themes were rechecked in the text for the coded extract. Themes were then defined and named and, in contrast with the immersion phase of the data in step one, ‘distance’ from the data was needed in this stage to maintain a critical approach towards the data analysis and examine the precision of the coding process. Following distancing from the data to ensure congruence and sensitivity, the final themes were verified.

## Results

### Characteristics of the included studies

Of the seven papers included in this review they were published across five different journals from 2012–2019 (2012 *n* = 1; 2013 *n* = 2; 2016 *n* = 1; 2018 *n* = 2; 2019 *n* = 1). Four papers were published in PC journals: International Journal of Palliative Nursing *n* = 2 [28, 29], the British Medical Journal of Supportive and Palliative Care *n* = 1 [30], and the Journal of Palliative and Support Care *n* = 1 [31]. The remaining three papers were published in wider topic journals such as the International Journal of Qualitative Studies on Health and Wellbeing *n* = 1 [32], Text Content Nursing Florianopolis *n* = 1 [33], and the Journal of Clinical Gerontology *n* = 1 [34]. Papers originated from United Kingdom (*n* = 3) [28–30], Canada (*n* = 1) [31], Denmark (*n* = 1) [32], Brazil (*n* = 1) [33] and the United States of America (USA *n* = 1) [34].

The identified studies provide a broad perspective of nursing in a specialized PC inpatient unit across several international countries. Of the hospice/palliative care units only two studies identified the number of specialist PC beds, Boa et al. [29] utilized a 24 bedded hospice in Scotland (UK) and Lavoie et al. [31] utilized a Canadian 15 bedded hospice. The other studies utilized hospice/palliative sites, but bed capacity was not reported [28, 30, 32–34]. Norwegian researchers [32] utilized two hospices in Denmark one of which was close to a large city and the other in a rural area. In Brazil researchers [33] utilized a centre for PC in a public teaching hospital, in the UK researchers, utilized a hospice [28, 30], and in the USA [34] utilized three hospices.

Of the seven studies included in this review, six used qualitative methods where designs included: phenomenology [32, 34], case study design [29, 33], qualitative descriptive [28], pre- and post-process design [31] and the remaining paper was a systematic qualitative review [30]. There were numerous sampling methods utilized in keeping with a qualitative design and some studies included other members of the Multidisciplinary Team (MDT) within the specialist PC unit [29, 31]. Following data analysis, the final themes identified were enhancing patient care (1); being with (2); exposure to suffering and death (3), and nursing values seen but not heard (4). It is inevitable given the topic of this review that there was some overlap between the themes and sub-themes) and this highlights the all-embracing nature of nurses working in inpatient SPCUs role (Table 2).

**Table 2:** Codes, subthemes and themes

**Codes**	**Subthemes**	**Themes**
Individualized care; person centred, decision making; assessment of need; goal setting; who sets the goals? patient goal setting; individual disciplines set goals; seeing the person not the patient; risk assessment; hearing the patients voice; knowledge; competence	Professional led care	**1. Enhancing patient led care**
	Patient centred care	
	Partnership care	
Being truly present; building relationships; being emotionally touched; knowing the patient; presence; sharing the journey; spending time; being with; listening; respect; knowledge, competence; listening; holding the silence; seeing the person not the patient	Building intimacy and report	**2. Being with**
	Developing skills & strategies enabling true presence with the patient	
	Being emotionally touched	
Finding meaning in death; self-care; making sense of what's happening; talking to colleagues; having a purpose; professional development; being emotionally touched; competence; knowledge; education; living life; making the most of life; looking after yourself; watching out for colleagues	Exposure to suffering & death can be stressful	**3. Exposure to suffering and death**
	Finding meaning in death allows opportunity for growth	
	Maintaining personal integrity	
More than just basic care; Extraordinary care; listening; responding; Caring; touch, emotionally touched; Presence; being with; self-care; personal integrity; making a difference; touch; kind; caring, empathetic; self-awareness; self-knowledge; reflection; holding the silence	Making a difference – from routine care to something more	**4. Nursing values seen but not heard**
	Emotional intelligence	
	Behaviours	

### Enhancing patient-led care

This theme describes how nurses working in inpatient SPCUs endeavored to know and understand their patients’ values and wishes, respecting where each patient is at in their journey and how the patient experiences both their illness and their care. Patient-led care impacts the achievement of a shared vision of patient- centred and patient-led care that is individualized and holistic. Four studies highlight how multidisciplinary professionals in PC led goal setting for patients and this was described as professional-led care [28, 29, 31, 33]. Goals ranged from everyday practicalities such as who washes the patient or can the patient go for a walk to decisions regarding symptom management [28, 29, 31, 33]. Boa et al. [29] described nurses working in inpatient SPCUs using a risk assessment-based goal-setting process and while the other studies did not specify tools the nurse was focused on assessing risk. For example, the patient wishes to wash him/herself, which he/she can do in bed with nurses present for support and assistance, but they {the nurses} wash the patient, possibly because it is quicker [31] illustrating task vs patient- centred care, this occurs when the nurse puts his/her need to get the task done before the patient wishes. This created a dichotomy between patient care led by professional expertise as opposed to patient values and or choice [31]. There is a fine balance here as patients look to the professional for expertise and guidance [34]. However, professionals need to ensure that they are not reducing opportunities for independence as their efforts become task orientated to ‘get the job done’ [29, 31]. Listening to patients and respecting their goals and wishes becomes key in PC [28–30] and based on listening personalized care develops [28, 31]. Here patients receive care ‘the way they want’ which supports autonomy, a patient- centred approach to human becoming and the philosophy of PC [31]. This is represented in moving from “letting them (the patient) do” to ‘being with them (the patient) while they do’ [33]. Enabling patient autonomy is achieving by considering the patients’ way of understanding the world and the choice he/she makes based on the individuals’ beliefs and value system [33]. However, with autonomy comes risk and this conflict was encountered where professionals anticipated risk to the patient while supporting patient wishes [29].

Within practice, this conflict is under review, and from a series of lectures and workshops which were based on Parse’s human becoming theory [35] participants in Lavoie et al.’s study [31] moved from suggesting measures to favor patient wellbeing and risk reduction in professionally led care to patient-led care wherever possible. This caused nurse participants to become more inclined to listen and set goals according to the patients’ needs and wishes [28, 31]. For such a process to occur the nurse must be truly present with the patient and listen to the patient’s needs or goals [28, 33], and, through this process making the invisible art of listening visible [31, 33]. However, it is recognized that nursing care does not occur in isolation, and care is enhanced by collaboration and team working [29, 29]. This partnership in care refers to collaborative care between the patient and the health care professional such as the; partnership between patient and nurse [33], partnership between nurse and patient [31], and partnership between patient and MDT [29]. This suggests that adopting a patient- centred approach enables nurses to be respectful and responsive to patients’ preferences, values and beliefs and thereby increasing patient choice and participation in their care [28, 29, 31, 32]. Conversely, the position of patients within the team or collaboration is not discussed and the focus is on professional-led care [28].

### Being with

Six of the seven studies identified ‘being with’ or ‘sharing the journey’ [28, 30–34], and building intimacy and rapport are key aspects within this process [31, 32]. Nurses must get to know their patient and their unique character through the development of a therapeutic relationship and this is a form of building intimacy so that the patient senses a safe and trusting presence in the nurses working in inpatient SPCUs who understands their needs and is truly present with them in their journey [31]. Essential within this relationship is acknowledging the patient’s emotional reactions to death and dying [32] and for the nurses working in inpatient SPCUs to allow themselves to be ‘emotionally touched’ by patients [30, 32]. However, Powell et al. [30] and Ingebretsen et al. [32] offer a note of caution so that emotions would not become overwhelming and there is a continuous balancing act between nurses’, patients and their feelings, and this can be simultaneously draining and enriching [30, 32]. Being with the patient is considered part of holistic care and presence with the patient is “not about doing, it’s about being” [28].

Although not easily measured or visible there was a value for the elements of care, compassion, and commitment, and these were reinforced in areas such as: touch [33], being with [28, 33], building intimacy, and rapport [31, 32]. These aspects are hidden values that are redefined within specialist PC units [33]. Length of stay in a SPCU for a patient can enable the development of a relationship that allows the nurse to gain a better understanding of the patient and therefore emotionally connect and empathize with them [28, 34]. For nurses to be able to maintain this connection they must develop strategies and skills in preserving personal integrity thus enabling true presence with the patient [30]. This involves managing one’s own emotions but also the emotions of others (patients/colleagues) daily [30]. Such strategies consist of both relational and technical aspects where nurses will retreat behind their uniform which allows them to move from ‘being with’ to ‘doing to’ to protect themselves [30] or having a professional approach as opposed to a relationship approach [30]. To achieve true presence and protect personal integrity the nurse must find a balance between the two and move beyond “the strategy of wearing a coat” [32].

### Exposure to suffering and death

Nurses working in inpatient SPCUs are regularly exposed to death, dying, and suffering as a core component of their work which can be stressful [30, 32]. The exposure to death was intense and challenging [32] and the two most significant concerns that nurses considered stressful were when patients suffered uncontrolled symptoms [32] and patients presenting with underlying mental health issues [30]. As a consequence of working closely with death and dying some nurses accept death as a natural element of life [32] and nurses feel honored because of their opportunity to spend time with people in the last part of life and this experience was perceived to be both personally and professionally enriching [30, 32]. ‘Being able to see the world through the eyes of a person who will soon be gone’ was seen as ‘a privilege, to observe the world with eyes that are not mine’ [32]. Thus, nurses working in inpatient SPCUs described their work environment as a ‘life confirming’ place, more centred on life [32] and as a result they no longer deferred things that they desired to do in their own life. They explained that closeness to death provided them with a greater perspective of how they wanted to live their lives and what was important [30].

The intertwining of personal and professional self contributes to nurses feeling well equipped and reassured in their role as professionals [32]. However, some nurses use strategies that are technical or relational, which allow the nurse to express or suppress their emotions [30, 32]. Technical busyness is concerned with the technical and/or administrative aspects of the role which takes the nurse away from the patient in terms of time and physical presence [32]. One such strategy is where nurses use the ‘white coat’ or instrumental touch as protective mechanisms behind which the nurse can hide from the reality of the moment [32]. Other strategies that nurse’s utilize, is the giving and receiving of support and this may be through clinical supervision, open discussion or emotional debriefing and maintaining a healthy work/life balance [28–30, 32–34].

### Nursing values seen but not heard

This theme describes the essence of expert care provided by nurses working in inpatient SPCUs and although nursing values were implicit within the nurses’ voices in the studies, the concepts of compassion, caring, and commitment were unspoken. The shift from task-oriented care to patient- centred was evident [31] and nurses express that understanding the patient is fundamental so that the nurse can harmonize with the patients’ rhythm to optimize their comfort and needs [31, 33].

Nurses have the potential to advance intuitive nursing into routine nursing practice [30] and build relationships while nursing one’s patients enable nurses to be personalized and proactive as opposed to reactive [34]. In the business of everyday practice, ‘being with’ can be achieved through attention to detail, active listening, and touch, all of which are essential elements of routine nursing practice but ‘something more’ in specialist PC [33]. Nurses who cognitively process their experience by talking and trying to make sense of it, prepare themselves for ongoing challenges in their clinical practices [30]. Within this process the individual needs to acknowledging their emotions and be aware that these emotions can be both enriching and draining at the same time [32] and not be overwhelmed by these emotions [32]. Furthermore, nurses must pay attention to their gestures and attitudes, the value of touch, staying silent or holding someone’s hand, giving support, and showing kindness are described as exemplary behaviors for nurses in PC [33]. Also, nurses’ approach to difficult conversations is associated with skills and behaviors which aim to alleviate suffering and these can be defined as compassion and care [28].

## Discussion

**Fig. 2:**
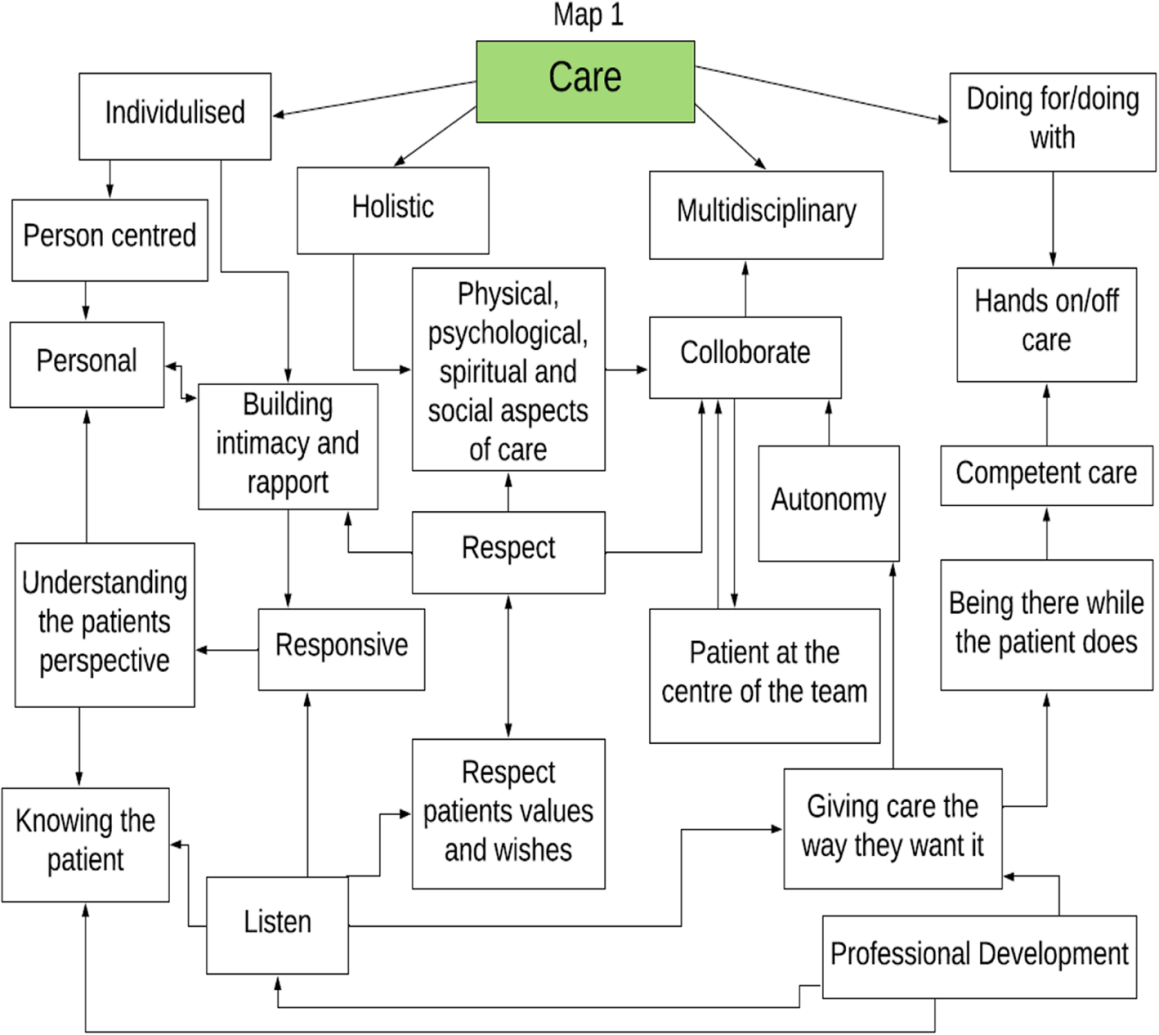
Care thematic map

**Fig. 3:**
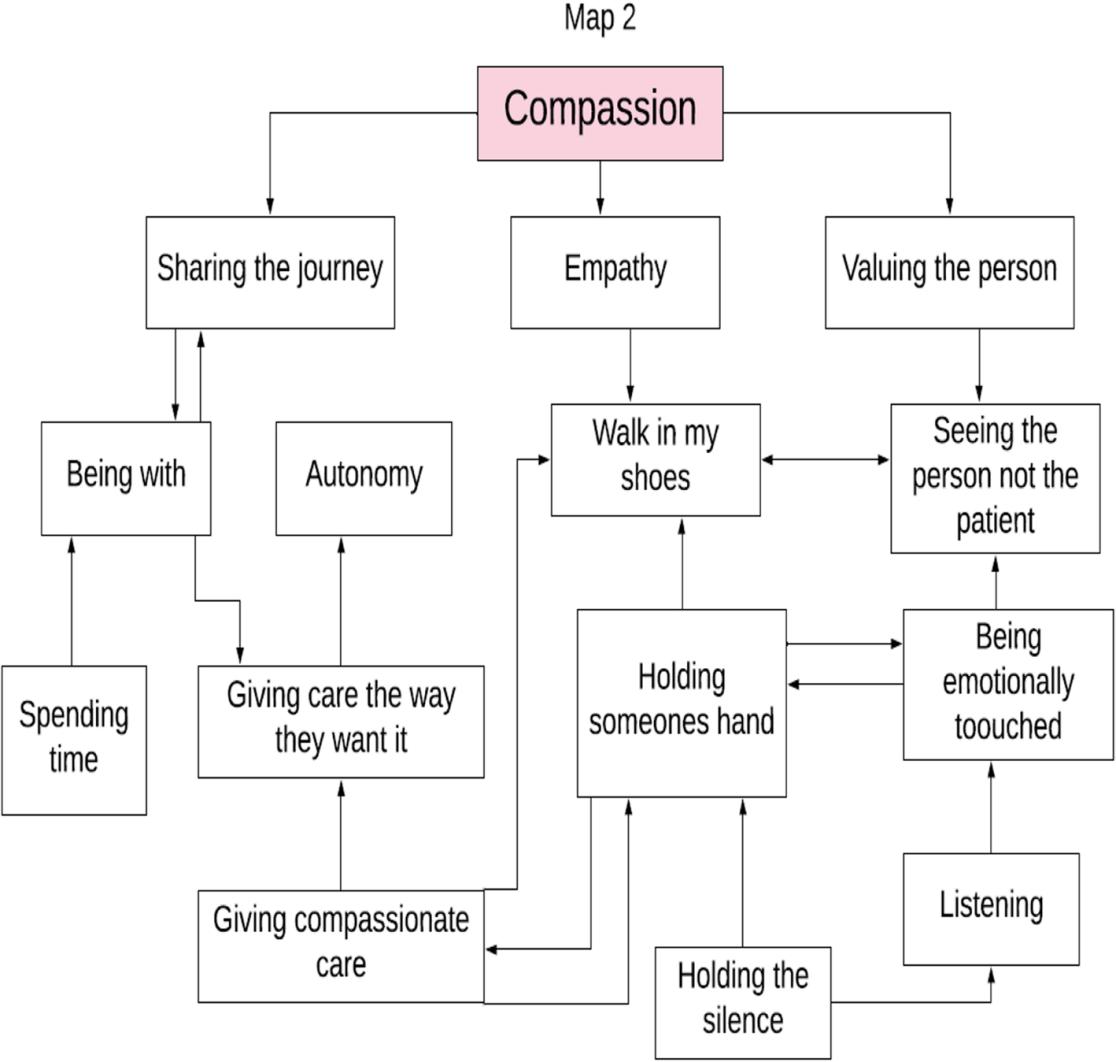
Compassion thematic map

**Fig. 4:**
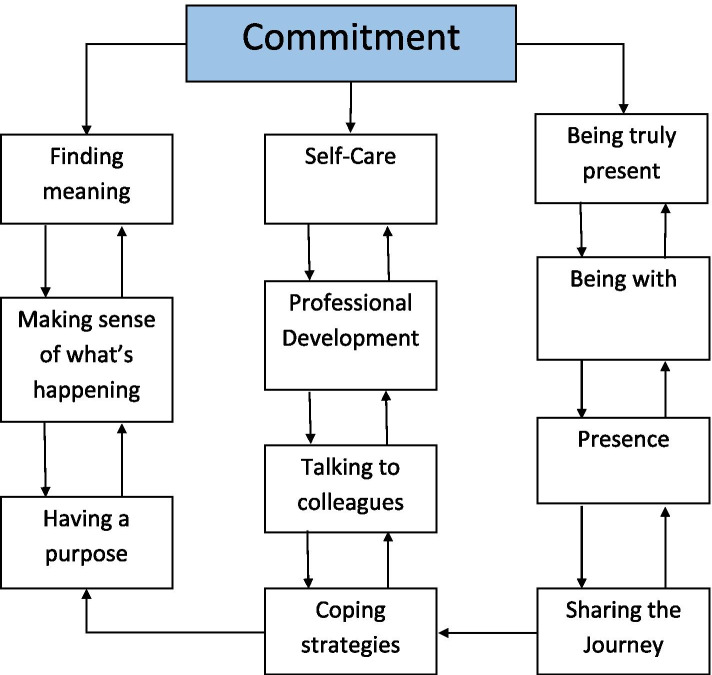
Commitment thematic map

In this review the actions, thoughts, emotional intelligence, and behaviors of nurses working in inpatient SPCUs in the reviewed studies all demonstrate the core values of nursing; care, compassion and commitment, and while depicted within descriptions of practice they were not articulated (seen but not heard). Unfortunately, in the days of evidence-based practice and professional accountability, unidentified work becomes unproven work that is eventually devalued and invisible and PC nursing is at risk of being a vague and non-descript part of the multidisciplinary’ team [9, 35]. The results of this review highlight the fact that the values of nursing while seen are not always heard and to make visible the values identified within this review the researchers created a thematic map for each nursing value (Figs. 2, 3 and 4). This thematic map utilizes the words used by participants within the studies reviewed to make visible the values of nursing in palliative care and guide the documentation within practice. Furthermore, a combined representation of the three thematic maps was developed (Supplementary file 2) to illustrate the values and the fact that the concepts within the values are fluid and can overlap across values. The findings of this review highlight the need to focus on how nurses working in inpatient SPCUs are making each of these values visible and the challenges in providing measurable evidence of patients need and wish for artistic, intuitive, and nursing skills to receive equal recognition with that of the scientific-technical elements of nursing that are more easily seen and recorded. However, through exploring a model that captures the values of nursing, nurses can be enabled to make visible the invisible and truly value what is often unseen as less important elements of their role. The thematic maps developed from this review may afford an opportunity to build on previous models of supportive palliative care such as Davies and Oberle [36] and Newton and McVicker [37]. The provision of nursing in a SPCU is currently challenged as the caring work of nursing has been hidden behind the gloss of scientific symptomlogical research. This may be as a result of the fact that caring work of PC nursing is private and intimate between patient and nurse and is not discussed with the wider team member [9]. Hands-on care, observational skills, and the intuitive ability to sense that a patient is distressed are considered expert nursing skills [38] but remain invisible to other healthcare professionals [35]. The findings of this review are consistent with other PC nursing literature [7, 9, 39] in highlighting the invisibility of the essence of caring.

Caring is a fundamental value underpinning palliative nursing, treating the patient as an individual as opposed to a condition or disease, and the value that both patients and nurses place on this cannot and should not be underestimated [40–42]. Recognizing this Kitson et al. [43] argue that as opposed to depersonalized and task-orientated care that is emerging within healthcare, “nursing must now reclaim and refine the fundamentals of nursing care.” As nurses working in inpatient SPCUs continue “being there” and “doing for” patients and combining this with sensitive communication they demonstrate, anticipate, and resolve, in a ‘think and link’ approach to care [44]. For example, the nurses working in inpatient SPCUs is not only washing the patient in bed, but he/she is also engaging with the patient and talking, observing, looking at their family photos, building a picture of the person, anticipating their needs, laughing, getting to know them, talking about their care, their family their hopes, dreams, and wishes. Knowledge gained through informal assessment concerning the patient and his/her family are internalized by the nurse and reflected on and revisited when planning/discussing patient care with the patient, family, and the MDT. This aesthetic aspect of nursing is frequently understated both in nursing documentation and in the literature and Larkin [39] argues the need for nurses working in inpatient SPCUs to develop a balance between ‘scientific clinical judgement’ and ‘artistic intuitive practice.’ Henry [45] agrees and argues that nursing is more than a set of tasks instead it is a way of ‘seeing, feeling, and knowing and to be an effective nurse he/she also needs to be an ‘art-full nurse’. Prior [46] in 2001 highlighted the value and comfort of nursing to the patient but also the complexity of nursing care and argued that nursing must demonstrate the value and impact on patient care so that it may be made visible, evidenced and recognized as a fundamental element of patient care. It is of note that 10 years later Haraldsotti [47] warned that practical tasks such as washing and dressing were being given priority instead of the psychological and emotional needs of patients and questioned how nursing can re-establish this basic but essential aspect of nursing care as complementary to patients’ symptom relief in specialist PC. It must be remembered that while attending to patient’s bodily care needs is considered an everyday occurrence for both nurses and the MDT in SPCUs, this is not the case for their patients as it is a private and intimate act which builds a bond between the caregiver and the receiver in which other intimate confidences may be made [48]. This notion highlights how bodily care provided to patients with care and compassion demonstrates the essence and complexity of nursing.

Compassion requires two different psychologies, firstly awareness and engagement, and secondly the skilled intervention with the action required. This corresponds with Carper’s ways of knowing—empirical, aesthetic, ethical, and personal [49] and proposes that nursing practice is the practical “knowing how” that the nurse has in a particular situation and which is used to achieve a particular result. This nursing action relies heavily on experience, scientific, and intuitive knowledge resulting in a higher order of nursing practice [50]. This higher order of nursing is congruent with Benner’s work from “Novice to Expert,” [51] for example an expert nurse may have many qualities, attributes and abilities and be able to incorporate several aspects of different models to provide a unique plan of care that bends and sways with the patients’ needs at that particular moment in time.

Even the briefest exchanges with patients can demonstrate a compassionate action and this is not new to PC nursing but significantly it is only discussed within the nursing team and not discussed with other interdisciplinary team members and not documented in patients’ notes as part of a care plan and therefore is rendered invisible. This has arguably allowed for science in nursing to dominate over the art and without the artistic aspect, nursing is in danger of becoming mechanistic with the nurse serving as little more than a technician [45]. From the literature it is clear that patients want more than a technician, patients want nurses committed to getting to know them and give of their time, understanding their experience and “how it feels {to be} in my shoes,” being more compassionate with sensitive and clear communications [42, 52–55]. Mohammed et al. [56] highlight that nurses through building their relationship with patients it allows them the opportunity to discuss very sensitive issues with their patients but at the same time knowing when to stop, but this expertise would be unseen by other healthcare professionals. This highlights artistic, compassionate aspects of nursing practice that have been invisible but fundamental to both the patient and nursing practice reinforcing the argument that nurses in their everyday routine work with the patient and family are doing something more. In this review, Ingebresten et al. [32] and Powell [30] conclude that nurses working in inpatient SPCUs demonstrate their ability to cognitively process their thoughts and verbalize their feelings and in so doing they make sense of the experience they are dealing with in a manner which can inform and enhance mastery in their clinical practice. The ability to be empathic, to stand back, think, and reflect on the care that is being given to patients is important [57] and aligns closely with compassionate care and the values of nursing described by Becker [38]. The “compassionate mind” [58] involves a combination of complex abilities and skills in conjunction with the attributes and qualities of the healthcare professional. However, while nurses’ express compassion in attending to the ordinary but essential needs of their patients, the clinical environment should be designed to foster such behaviors [7]. This raises the conundrum that unless these values of care, compassion, and commitment are articulated and recorded they cannot be recognized through audit and therefore remain invisible.

Professional commitment has been described as a predominant source of positive professional behavior in health care professionals and as such correlates significantly with the quality of patient care [59]. Commitment requires both an intention and an action and nurses’ actions and behaviors could be described as the art of what they do and how they do it. Action behaviors reflecting nursing values noted in this review include presence, listening, responding, being there [28, 29, 31–34]. Behaviors noted in other literature include sitting at the patient’s bedside, not standing over the patient, touching the patient, timely care, holding someone’s hand, smiling at the patient, hands-on clinical care i.e. washing, dressing [37, 44, 52–54, 60, 61]. In keeping with the findings of this review these action behaviors would be accompanied by compassionate behaviors including, respect, valuing, showing warmth, presence, paying attention, understanding, and empathizing.

The value of commitment is demonstrated in this review although again not articulated in the studies. This value can be seen in the concept maps (Figs. 2, 3 and 4, Supplementary file 2) to include concepts included previously in this discussion demonstrating the links and parallels between the values care, compassion, and commitment none of which stand alone. This sits comfortably within Carper’s ways of knowing [49] and results in a balance between the art and science of nursing [62]. Often it is the minute details of how it looks in practice that is missing [63, 64]. Part of this process is to develop and enhance nursing behaviors so nurses can “think and link” when delivering patient care through verbal and non-verbal behaviors [44]. Through this process nurses’ understanding, anticipating, and responding to patient care is not only scientifically evidence-based but also makes visible the intuitive art of nursing which has not heretofore been seen and therefore has gone unacknowledged [44, 65]. A further action demonstrating commitment is the need for self-care or maintaining personal integrity [30, 32, 66], developing coping strategies [29–34] and professional development in PC [28] were all described in this review and the wider literature.

Several models of nursing in PC such as Davies and Oberle [36], Newton and Vicar [37], Pfaff and Markaki [67], and Bao et al. [29] are designed to inform and support clinical practice. Sinclair et al. [53], recognizing that patients prefer a compassionate orientation (as opposed to sympathy or empathy) towards action and virtue-based motivator, developed a practice model based on the principle of a relational understanding of the patients’ needs and suffering supported by a compassionate action or behavior. However, the advancement of this, and similar models into everyday practice is complex and even with the support of practice development, the implementation of nursing research into practice is challenging [68]. It may be argued that Davies and Oberle [36] Supportive Care Model for PC Nursing highlights the unseen elements of nursing care that are being delivered in specialized palliative settings. The dimensions within this model; connecting, doing for, finding meaning, preserving dignity, and valuing, are closely associated with the nursing values of care, compassion, and commitment. A challenge of this model is that it is regarded as intuitive and difficult to evidence in practice [37] however in a review of the model [37] findings support its utilization in contemporary and specialized palliative nursing with the additional dimensions of displaying expertise and influencing other professionals. Larkin [39] suggests that the revised framework promotes an equal balance between the scientific and the artistic intuitive nursing practice that is palliative nursing. However, in the pursuit of nursing excellence, nurses must endeavor to demonstrate compassionate nursing interventions that are evidenced-based and combined with an artistic intuitive practice that ensures nursing care can be visible not only to patients and their families but also to the wider multidisciplinary team.

### Strengths and limitations

A strength of this review is the insight gained into the role of the nurse working in inpatient SPCUs in the provision of PC. However, it is recognized that the time frame may have eliminated articles that were relevant and could have added to the discourse. This review highlights the dearth of literature specifically relating to the values of nursing in PC and the role of the nurses working in inpatient SPCUs. Studies predominately featured the views of health professionals and generalists, given that PC is an MDT effort this was not unexpected. However, we must recognize that it is challenging to recognize the actions of nurses working in inpatient SPCUs if they are absent from research or reported by others from a distance.

## Conclusions

In this review, it is apparent that nurses are embracing the core values of nursing whilst continually striving to retain the strong traditions of caring for the dying in terms of preparation of both family and patient for this event and the provision of care during and following a death. However, the increasing scientific and technical demands of nursing practice within the changing face of PC has resulted in causing the unseen and underreporting of the artistic and intuitive actions. The consequence of this is that such work becomes unrecognized, undervalued, and ultimately under resourced. The findings of this review suggest that within specialist PC, the science and biomedical model is continuing to dominate over the artistic Askelpian traditions and nurses must accept some responsibility for this through their failure to identify and document their actions. To regain and sustain Cecily Saunders vision of PC, a recalibration is needed between the two, and PC nursing needs to look critically at what they are providing and questioning their service model for patients with life limiting conditions be it in a specialist palliative care/hospice model or a hospital model.

## Supplementary Information

**Additional file 1. **PRISMA Checklist.

**Additional file 2. **Thematic Map.

## Data Availability

Data used for analysis in this review are all extracted from the original published reviews and are presented in Table 1 (Data extraction table).

## Abbreviations

AMED: Allied and Complementary Medicine Database
CCAT: Crowe Critical Appraisal Tool
CINAHL: Cumulative Index to Nursing and Allied Health Literature
MDT: Multidisciplinary team
PC: Palliative care
PCNs: Palliative care nurses
PEO: Population-Exposure-Outcome
PRISMA: Preferred Reporting Items for Systematic Reviews and Meta-Analyses
SPCU: Specialist palliative care unit
SPCUs: Specialist palliative care units
UK: United Kingdom
USA: United States of America
WHO: World Health Organisation
