# Supplementary material for: An integrative review to identify how nurses practicing in inpatient specialist palliative care units uphold the values of nursing
Source: BMC Palliat Care. 2021 Jul 16;20:111. doi: 10.1186/s12904-021-00810-6 (PMC8285858; doi:10.1186/s12904-021-00810-6)
Supplement: Supplementary file 2 — Additional file 2. Thematic Map. [file 12904_2021_810_MOESM2_ESM.docx]

**Supplementary file 2**

**Thematic Map**
